# Two Essays on the Diseases of the Spine: 1, On Angular Curvature of the Spine, and Its Treatment; 2, On the Treatment of Lateral Curvature by Gravitation, Lateral Exercise, &c.

**Published:** 1844-07

**Authors:** 


ART. XXI.

*Two Essays on the Diseases of the Spine:* 1, *On Angular Curvature of the Spine, and its Treatment;* 2, *On the Treatment of Lateral Curvature by Gravitation, Lateral Exercise, &c.* By R. A. STAFFORD. —*London*, 1844. 8vo, pp. 92.

THE present treatise is "Part of the Jacksonian Prize of the Royal College of Surgeons, on the Injuries and Diseases of the Spine, with considerable alterations, corrections, and additions;" it consists, as may be seen from the title-page, of two parts, on each of which we shall make a few observations.

The essay on angular curvature of the spine occupies fifty-one pages, ten of which are devoted to cases; and in the remaining forty pages we have one of the best accounts of the disease that we have ever met with. An analysis of this excellent little essay would be quite out of place, as it does not contain anything new. Suffice it then to say that the etiology, pathology, and symptoms of the affection are detailed with clearness, precision, completeness, and brevity; while the directions for treatment are, in our opinion, extremely judicious. We know of no better or safer work on the subject to put into the hands of the student or junior practitioner. We forbear, however, from giving any extract from this portion of Mr. S.'s book, as its merit consists in the judicious use of known materials.

The second essay does not purport to be a complete essay on lateral curvatures of the spine; its title, indeed, indicates that it is merely intended to recommend a particular mode of treatment in this very refractory deformity; we have not, therefore, any right to complain that this essay is greatly less complete than the former one; for although many particulars, more especially connected with the pathology of the disease, are omitted, or imperfectly discussed; yet quite enough is said to illustrate and render intelligible the author's view respecting the treatment of the affection, to which we shall advert somewhat fully but yet briefly, inasmuch as Mr. Stafford, in the advertisement or introduction on the flyleaf, says "In lateral curvature I flatter myself I have simplified and offered an original method of treatment,—a method which at once takes these cases out of the hands of the charlatan and pretender, and places them in the power of the profession at large. I have only to add, that my experience has proved the utility of the plan adopted."

Mr. Stafford attributes lateral curvature of the spine: 1. To weakness of the spinal column and its appendages, (pp. 53-64.) 2. To the muscles of one side of the spine being brought into greater action than those of the other, whether from one limb being used more than its fellow, or from an habitually faulty carriage tending to deviate the trunk from the perpendicular. (pp. 53-8.) 3. As a cause secondary to either or both of the foregoing, to the centre of gravity being thrown on the edges of the vertebra, a circumstance which tends to aggravate the curvature, and on which, in the author's opinion, too little stress has been hitherto laid. (pp. 56-7.) Indeed, if we understand Mr. Stafford right, this secondary cause becomes ultimately by much the most important of all; muscular action, according to his views, can never do more than *commence* the curvature, which is subsequently aggravated by the unequal distribution of pressure on the vertebræ; for Mr. Stafford thus appeals to the condition of the muscles on the *concavity* of the curvature in evidence of the great influence of the secondary cause in question; those muscles, he says, "must necessarily be contracted *without having any power of action*, and thus the lateral movements of the vertebræ, as in other joints that are anchylosed from the same cause, is completely prevented. It would appear, then, when the curvature is completely formed, that the muscles which were first brought to pull the spine out of its centre of gravity, became rigid and contracted, while the opponent muscles on the convex side became stretched." (p. 57.) And again, "The muscles and the ligaments on the *concave* side of the curve are necessarily contracted, and, consequently, *are rendered incapable of performing their proper functions*, whilst those on the convex side are equally stretched beyond their natural tension, by which they are *also* in a greater or less degree *made useless.*" (pp. 62-3.) If, then, the muscles on the *concave* side as well as on the convex side have no "power of action" and are "made useless," once the curvatare is established clearly, muscular action can be but an initial cause of the deformity, and must, after a certain period cease to operate.

Mr. Stafford's recommendations for the treatment of lateral curvature are as follows: we need scarcely premise that he does not hold out any hope of success in aggravated cases of very long standing. In the first instance the general health must be attended to; and then we must restore "the muscles and ligaments of the spine to their proper functions, which can only be done by bringing them into use." (p. 65.) If on inquiry the deformity seems referrible to "weakness of the spine alone, our chief care should be to relieve it of the weight it has to sustain, which can only be done by lying down," (p. 65;) but the recumbent position must alternate with as much daily moderate exercise as the patient can bear. If the habit of standing on one leg, or of using one shoulder more than the other, has caused a tendency to deformity, the habit must be corrected, and also the opposite limb should be called into frequent action, by means of some suitable game or exercise, and general gymnastics, appropriate to the particular case, should be enjoined. Such means may suffice in simple or commencing lateral curvature, but when the distortion is confirmed, then Mr. Stafford has derived the greatest benefit from *lateral exercise.* "It was from side to side that the curve first commenced; and consequently, to bring it back again to its natural state, that set of muscles ought to be more particularly acted on which would effect this object. The muscles and ligaments on the side of the spine are those which have chiefly lost their function, and to restore them, therefore, should be our chief aim." (p. 70.)

In order to perform this lateral exercise, Mr. Stafford has devised a machine, which consists of a semicircular wooden frame, resembling the platform or rocker of a hobby-horse. This frame lies on the ground on its convex surface, and the ends of a rope, which passes through two pulleys fixed in the ceiling, at a distance from each other equal to the length of the rocking-frame, are attached respectively to a bar at each end of the frame. "The patient stands upon this machine, taking hold of the rope by each hand, and then rocks himself or herself backwards and forwards (from side to side, rather,) by which both the lumbar and dorsal curve are acted on laterally." (p. 71.) Mr. Stafford has "hardly known an instance when it (lateral exercise) has not been of the greatest service." (p. 70.) But "lateral exercise, however, will not always recover a lateral curvature. The spine is ometimes so completely distorted and the vertebral column so entirely thrown out of the centre of gravity, that the muscles have lost their power. They are so stretched on the convex, and so contracted on the concave side of the curve, that they cannot act. In such cases lateral exercise will not alone be sufficient. More must be done—the spine itself must be elongated; and the best method of accomplishing this is by gravitation of the body. To effect this object I have invented a machine by which the patient can be raised up from the ground by the upper part of the body, while the lower part hangs suspended. Hence the lower part, by its own gravitation, and by additional weights being hung round the hips, gradually elongates the spinal column, until it becomes nearly if not quite, for the time being, straight. In this manner ........ the muscles on the concave side are lengthened, while those on the convex are shortened, and allowed to contract, whereby they are both put into a more favorable position to pull back and retain the vertebræ in their situation." (p. 77.) "After the machine has been used I usually recommend the lateral exercise, as the muscles and ligaments are then in the best state to be strengthened." (p. 79.)

The machine here mentioned is called by Mr. Stafford the "spine elongator." We refer the reader to the original essay for a description of the apparatus; but numerous contrivances have been already proposed to effect the same object.

Several cases are detailed—no doubt quite accurately—in which the treatment we have described was adopted with perfect success. In one case, where "the curve of the spine was so great, that the spinous processes were actually hid under the right scapula or blade-bone," &c. (p. 81;) the spine ultimately "deviated so little from its natural course, that no one could ever have perceived that there had been a deformity." (p. 82.) In another similar case the patient, after two years of treatment, "had a beautiful figure." (p. 83.) Such success in such cases is, we must confess, new to us; and we could urge not a few theoretical objections to Mr. Stafford's practice, founded on his own views. We might, for example, say that, in confirmed lateral curvature, the muscles are, on Mr. Stafford's showing, inoperative in keeping up and increasing the curvature. How then can restoring their action remove a condition of things of which they have ceased to be the cause? and, least of all, how can "lateral exercise" accomplish our object? for it *equally* exercises the muscles on *both* sides of the spine, and restores their functions equally. But how can restoring the action of the muscles on the concave side of the curvature remedy the deformity? for it was those very muscles that originally produced the deformity, which their active contraction must tend to increase. We waive, however, this and other such speculations. Much as we respect theory in surgery, practical facts cannot be questioned; and we therefore leave Mr. Stafford's views and practice to be tested by experience, earnestly hoping that he may not have been misled by some of those unaccountable mistakes into which the most acute observers and conscientious men have fallen when advocating a favorite practice, and especially if that practice presents any novelty. This leads us to observe that Mr. Stafford's practice is new, so far as regards the point of lateral exercise and the machine he has devised for its performance at least; we certainly do not recollect any previous proposal to call the muscles of the spine into action in this particular direction. It just occurs to us to add that we are the less willing to theorize respecting Mr. Stafford's practice, as the condition of the muscles on the concave side of a lateral curvature of the spine is still an unsettled point in pathology; according to some they are in a state of active contraction: others maintain that they are passively contracted; while others say that they are atrophied, and all appeal both to clinical facts and to dissections in support of their views. We must, however, now conclude by recommending Mr. Stafford's Essay for the perusal of our readers.

---

